# Study of Traffic incidents with much injured in Kermanshah province from 2012 to 2018

**Published:** 2019-08

**Authors:** Touraj Mohamadyari, Mahnaz Mohamadkhani, Saeb Modaresi, Shahram Ekhtiari

**Affiliations:** ^*a*^Master of Science in Community Health Nursing, Emergency Center of Kermanshah (EMS), Kermanshah University of Medical Sciences, Kermanshah, Iran.; ^*b*^General practitioner, Emergency Center of Kermanshah, Kermanshah University of Medical Sciences, Kermanshah, Iran.

## Abstract

**Background:**

Traffic accidents are one of the problems and threats to the health of people in the community. This study was performed, in view of the increasing number of accidents and casualties caused by it, with the aim of investigating traffic incidents involving crashes with more than 4 injured people, Accidents with more than 2 death Announced to Kermanshah University of Medical Sciences EOC unit from 2012 to 2018.

**Methods:**

This study is a retrospective descriptive study. The research sources included 1382 reports of traffic accidents in Kermanshah province that in the form of A-9 information, all of the medical emergencies and provincial-level hospitals were collected from the Ministry of Health from traffic incidents from 2012 to 2018.

**Results:**

The results of this study showed that 1382 incidents reported during the last 7 years Except for 2017, has increased. In total, the highest number of accidents occurred in the month of September and then the month of April and the lowest in May. During this period, 8425 people have been traumatized that 8.45% of them were reported death. On average, 1076 injured were transferred to the hospital and 102 deaths were reported before an ambulance arrived. Pride has been involved in 42% of the incidents. Most accidents occurred at around 14:00.

**Conclusions:**

Since the incidents and deaths, injuries and disabilities increasing caused by these incidents, Therefore, preventive proceedings and proper planning to reduce these incidents seem necessary.

**Keywords:**

Incident, Accident, Traffic accidents, Kermanshah province

